# When Are Mobile Phones Useful for Water Quality Data Collection? An Analysis of Data Flows and ICT Applications among Regulated Monitoring Institutions in Sub-Saharan Africa

**DOI:** 10.3390/ijerph120910846

**Published:** 2015-09-02

**Authors:** Emily Kumpel, Rachel Peletz, Mateyo Bonham, Annette Fay, Alicea Cock-Esteb, Ranjiv Khush

**Affiliations:** 1 The Aquaya Institute, Nairobi 00505, Kenya; E-Mails: rachel@aquaya.org (R.P.); mateyo@aquaya.org (M.B.); annette@aquaya.org (A.F.); alicea@aquaya.org (A.C.-E.); 2 The Aquaya Institute, Larkspur 94939, CA, USA; E-Mail: ranjiv@aquaya.org

**Keywords:** mobile phone data collection, sub-Saharan Africa, water quality, water monitoring, health agencies, developing countries, water utilities, information and communication technologies (ICTs)

## Abstract

Water quality monitoring is important for identifying public health risks and ensuring water safety. However, even when water sources are tested, many institutions struggle to access data for immediate action or long-term decision-making. We analyzed water testing structures among 26 regulated water suppliers and public health surveillance agencies across six African countries and identified four water quality data management typologies. Within each typology, we then analyzed the potential for information and communication technology (ICT) tools to facilitate water quality information flows. A consistent feature of all four typologies was that testing activities occurred in laboratories or offices, not at water sources; therefore, mobile phone-based data management may be most beneficial for institutions that collect data from multiple remote laboratories. We implemented a mobile phone application to facilitate water quality data collection within the national public health agency in Senegal, Service National de l’Hygiène. Our results indicate that using the phones to transmit more than just water quality data will likely improve the effectiveness and sustainability of this type of intervention. We conclude that an assessment of program structure, particularly its data flows, provides a sound starting point for understanding the extent to which ICTs might strengthen water quality monitoring efforts.

## 1. Introduction

Contaminated drinking water is a primary exposure route for fecal pathogens and chemical toxins, which are important global public health concerns [1]. Water quality monitoring is essential for evaluating contamination status, guiding protection and treatment strategies, and verifying management efforts. In many developing countries, national regulations specify institutional roles and responsibilities for drinking water quality monitoring [2,3]. Water quality monitoring includes operational and surveillance monitoring: operational monitoring is performed by water utilities to ensure the safety of their supplies while surveillance (or compliance) monitoring is performed by independent agencies that are usually responsible for public health (e.g., district or regional health offices, ministries of health) [1,4,5]. The effective use of monitoring data (e.g., water test results, water point maps, survey results), however, requires efficient information flows that reach all relevant actors, from the utility employees who implement protection and treatment activities to the administrators who establish management priorities and allocate resources. 

In-line sensor technologies are increasingly used by water suppliers for operational monitoring purposes to directly measure and obtain real-time data on certain water quality parameters [6]. Regulatory requirements, however, generally specify the use of laboratory-based diagnostic tests for parameters such as microbial contamination. This study focuses on data collected using laboratory methods. Mobile phone applications for improving information flows in the water and sanitation sector have included tracking water system performance, ‘crowd-sourcing’ information on water supply functionality, and reporting operational data from small, dispersed water supplies [7,8,9,10]. In a previous effort to improve water quality monitoring data management, the Water Quality Reporter (WQR) mobile phone application and a corresponding backend web service, the Water Quality Manager (WQM), were developed under the Aquatest water quality diagnostics program [11]. WQR is a Java 2 Platform, Micro Edition (J2ME) application with form-based data collection that allows water testers to submit data to a central database; WQM is a companion application that allows managers to view reports, visualize testing results, and establish contamination alerts [12,13]. 

The WQR/WQM system was field-tested in four water quality monitoring programs, including rural operational and surveillance monitoring in South Africa, rural surveillance monitoring in Mozambique, rural operational monitoring in Cambodia, and urban operational monitoring in Vietnam [12,13]. The field tests showed that the extent to which the WQR/WQM system influenced information flows was largely determined by existing data management structures. For example, while WQR/WQM improved the efficiency of data transmission in all four field-test settings, it only improved the availability of information within upper administrative levels in Mozambique; in the other three locations, managers already received test results via logbooks and email prior to the introduction of WQR [12]. These results imply that the applicability and success of mobile phone-based water quality data management systems are dependent on context and will vary according to the monitoring program structure. 

We identified water quality monitoring program structures most likely to benefit from mobile phone-based data management by analyzing information flows within regulated water quality monitoring institutions in Africa. We then describe a data management system that we tested with the Service National de l’Hygiène (SNH) of Senegal, an institution with a program structure that appears suitable for mobile phone-based tools.

## 2. Methods

### 2.1. Study Sites

We collected information on water quality monitoring activities from institutions responsible for regulatory operational and surveillance monitoring across Africa that were involved with the Monitoring for Safe Water (MfSW) capacity building program [14,15]. Initially, 72 institutions from 10 countries submitted monitoring information in their applications for participation in MfSW. Subsequently, 26 institutions from six countries (Ethiopia, Guinea, Kenya, Senegal, Uganda, and Zambia) were selected for the MfSW program. These included 11 water suppliers (two national suppliers, two regional suppliers, one private water operator association, and six individual municipal suppliers) and 15 surveillance agencies (one national health ministry, three regional surveillance labs, and 11 district health surveillance offices) that together were responsible for monitoring 118 urban water systems and 343 public health districts.

We developed a mobile phone application for managing water quality data with SNH, the unit of the Ministry of Health and Social Action in Senegal that is responsible for monitoring drinking water supplies. SNH field agents tested the mobile phone application in the regions of Dakar (the urban capital with an estimated 3 million residents), Diourbel, and Kaolack (both rural with estimated populations of 1.5 million and 1 million, respectively).

### 2.2. Data Collection

We obtained qualitative and quantitative data related to water quality monitoring activities between November 2012 and February 2015 during five stages of the MfSW program (Table 1): (1) applications; (2) needs assessments; (3) midterm assessments; (4) ongoing testing; and (5) testing of the mobile phone application (for SNH only). MfSW applications consisted of written surveys and one year of retrospective water quality data submitted by institutions. During needs and midterm assessments, we conducted in-person semi-structured interviews and observations with program managers and implementing staff. In the ongoing testing stage, institutions tested water quality and submitted water quality data monthly. We documented monitoring challenges through email and telephone communications. We analyzed implementation of the mobile phone application through in-person semi-structured interviews, observations, communication notes, and water quality testing data. We submitted the study protocol to the Western Institutional Review Board (WIRB) (Olympia, WA USA) for ethical review and received a determination of exemption from full review under 45 CRF 46.101(b)(2) of the Common Rule.

**Table 1:** Data collection.

Activity	Number of institutions	Timings (m/y)	Duration	Data Collection Method
Application	72	12/2012–5/2013	Minimal	Written application, one year retrospective water quality data
Needs assessment	42	12/2012–8/2013	1–5 days	Written survey, interviews, facility observations
Ongoing testing	26	7/2013–12/2014	3–16 days	Monthly submission of water quality testing data
Midterm assessments	26	3/2014–12/2014	2–7 days	Written survey, interviews, bservations
Mobile application test	1	7/2014–2/2015	2 months	Interviews, observations, testing data

**Figure 1:**
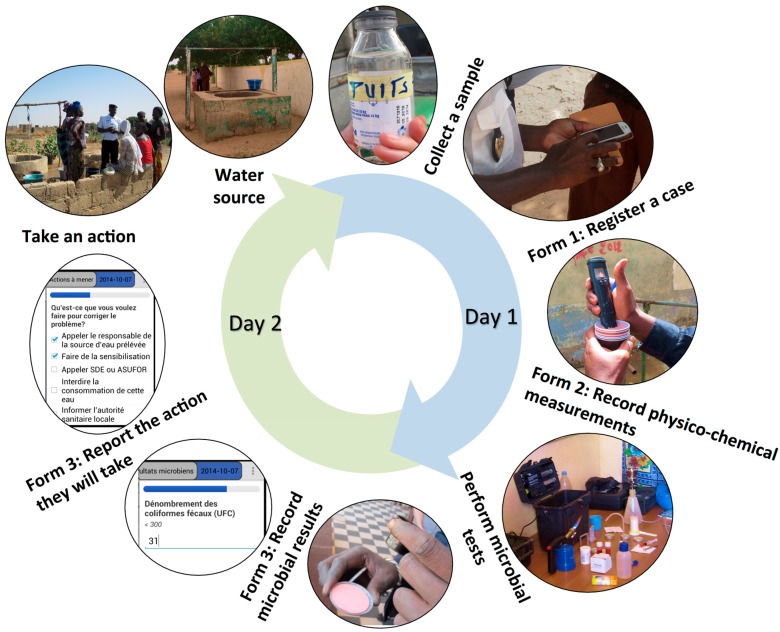
Process flow of the three CommCare forms used over two days by the Service National de l’Hygiène (SNH) to collect data about a water sample. A case refers to a single water sample.

In our collaboration with SNH, we initiated water monitoring data collection with a paper-based survey. Subsequently, we paired each of their 13 water testing kits with a mobile smartphone (Samsung Galaxy Fame S6790/S3 Lite) running on the Android operating system, which carried a data collection application that we developed using the CommCare survey management platform (Dimagi 2014; the application is available for download at www.commcarehq.org/exchange). We improved the application through field-testing between July-October 2014. SNH health agents entered the following data into the data collection application about a single sample using three forms filled out over two days (Figure 1):

- Form 1: Field Data Collection—GPS coordinates, water source, sanitary conditions, and water quality parameters tested on-site when collecting a sample.
- Form 2: Office-Based Physico-chemical Measurements—water quality measurements performed in offices the same day as sampling.
- Form 3: Microbial Testing Results and Action Reporting—fecal coliform colony counts, quality control procedures, and actions taken in response to contamination recorded the day after sampling.

### 2.3. Data Analysis

We analyzed quantitative data, including the number and frequency of water quality tests conducted by institutions, using the R software package (R Core Team 2014). Qualitative data, including interviews, observations, written surveys, and communication notes, were entered, coded, and accessed using NVivo software (QSR International). We performed queries through NVivo to identify data collection and management challenges and current use of ICTs in existing programs. Based on the qualitative data, we constructed full Data Flow Diagrams (DFD) for 12 of the final 26 MfSW program participants and simplified descriptions of data flows for the remaining 14 to depict each step in data collection, collation, and transmission [16,17]. We used qualitative and quantitative data and DFDs to develop classifications, or typologies, of program structures for the 26 program participants. 

## 3. Results and Discussion

### 3.1. Typologies of Water Quality Monitoring Programs

As depicted in the DFDs, we observed that regulated water quality monitoring programs follow a general framework. Individual ‘collectors’ (e.g., community health volunteers, laboratory technicians) visit a water source or a household and collect a water sample. They then transfer the sample by foot or a vehicle to a central ‘testing location’ (a laboratory, health center, hospital, office, or home). ‘Testers’ analyze the water samples for microbial indicators of fecal contamination, with results available and recorded after a day of incubation. Our DFDs begin with sample collection and end when results are collated within an institution. Institutions may subsequently report results to external regulatory agencies or consumers, though we did not address these downstream information flows in this analysis. 

**Figure 2:**
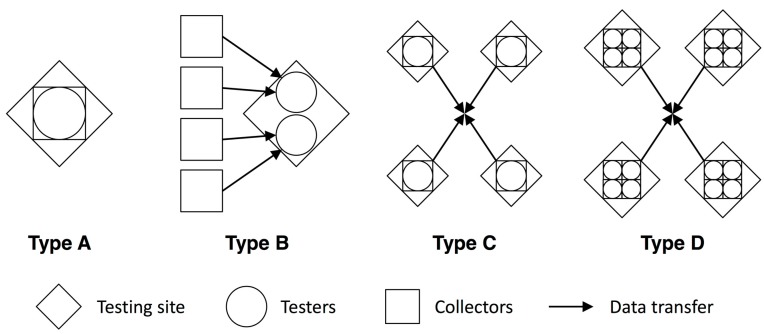
Generalized representation of typologies.

We used the DFDs to classify the MfSW participants into four monitoring typologies that are based on the following characteristics of an institution’s water quality monitoring program structure (Table 2 and Figure 2):

- the number of locations where testing is conducted (*i.e*., ‘testing locations’),
- the number of staff collecting samples and conducting tests for each testing location (‘collectors’ and ‘testers’)
- whether or not the same staff collecting samples also conduct the tests (‘collectors’ *vs.* ‘testers’).

**Table 2:** Typologies of water quality monitoring programs.

	A: All-In-One	B: Pass-It-On	C: Decentralized	D: Independent Teams
Number testing locations	1	1	>1	>1
Number collectors/testers per location	variable	>2 collectors, fewer testers	1–2 collectors and testers	>2 collectors, >2 testers
Staff collecting/testing	same	different	same	same
Number of MfSW institutions	12	4	7	3
Main data management challenge	Reliance on a few or one person	Data transfer from sample collectors to testers	Data collation and consistency between multiple testing locations	Data collation and consistency within and between multiple testing locations

#### 3.1.1. Type A (“All-In-One”): Single Testing Location, Collectors are also Testers

**Figure 3:**
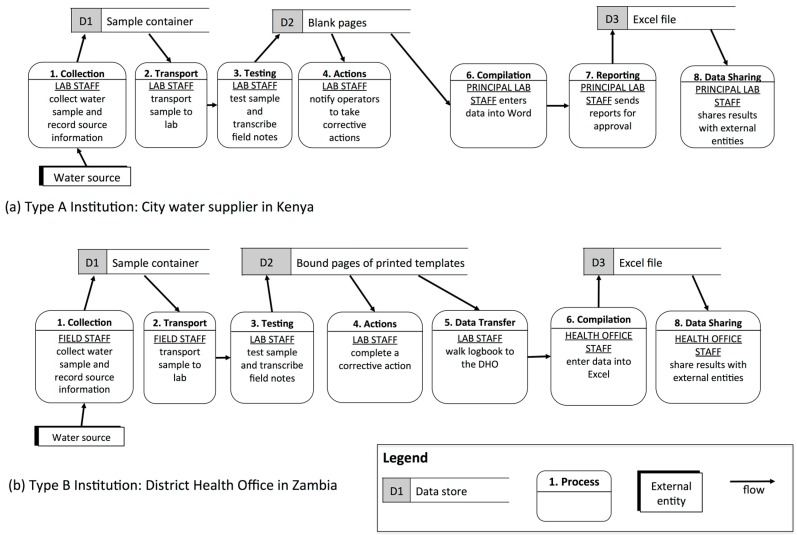
Data flows within Type A and B institutions shown through simplified data flow diagrams (DFDs).

In Type A institutions, the individuals who collect samples also test them at a single, central location. Each institution may have one, several, or many individuals collecting and testing samples. All institutions in this category had dedicated laboratory space for water testing. This typology was the most common and included water suppliers for individual towns in Kenya and Ethiopia, regional surveillance labs in Ethiopia and Uganda, and District Health Offices (DHOs) in Uganda and Zambia. For example, a water supplier in Kenya relied on two staff members to manage the testing program: these staff collected samples, transported the samples to the lab, performed the tests, recorded results in a lab notebook, and then entered them into a computer at the testing location (Figure 3a).

Typology A involves minimal data transfer and is, therefore, the least complicated of the four typologies. However, with only a few personnel managing the entire testing process, staff turnover has the potential to jeopardize monitoring and reporting activities. 

#### 3.1.2. Type B (“Pass-It-On”): Single Testing Location, Collectors differ from Testers

Type B institutions differentiated water sample collectors from testers who operated at a central location. Generally, there were many more collectors than testers. All MfSW partners included in this typology were surveillance agencies, including DHOs in Kenya and Zambia and a regional surveillance laboratory in Ethiopia. Two of these conducted testing in offices and one used a laboratory in the district hospital. For example, in a Zambian DHO, 15 Environmental Health Technicians (EHTs) from different health facilities collected samples and delivered them to a hospital laboratory. Laboratory staff received the samples, conducted tests, and recorded the results in a data logbook. The data were then transferred into an electronic database by DHO staff (Figure 3b).

The main challenge faced by Type B institutions lies in the transfer of information between sample collectors and testers. When collectors handed off water samples to testers, there was potential for poor coordination with the laboratory staff and for mixing-up samples and associated data (e.g., date, time, location, type of sample). One MfSW partner DHO with a large catchment area reported that sample collectors regularly arrived at the testing location with their samples to find that the testing staff had already left for the day.

#### 3.1.3. Type C (“Decentralized”): Multiple Testing Locations, Fewer Collectors and Testers

**Figure 4:**
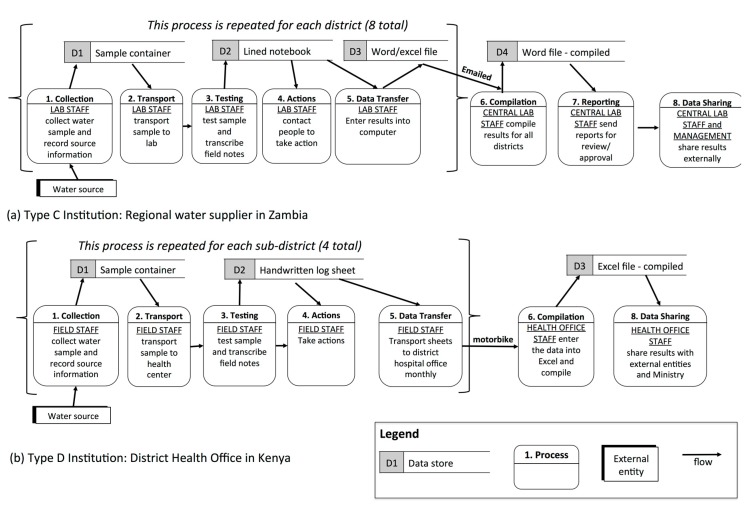
Data flows within Type C and D institutions shown through simplified data flow diagrams (DFDs).

Type C institutions have multiple testing locations, each with one or two individuals who both collect and test samples. All of these institutions analyzed samples in laboratories or with portable test kits based in offices. Data from all testing locations was periodically collated at a central location. MfSW participants in this typology included national water suppliers in Uganda and Guinea, regional water suppliers in Zambia, private water operators in Uganda, and DHOs in Zambia and Kenya. For example, a regional water supplier in Zambia had lab technicians in eight districts conducting water quality monitoring (Figure 4a). In each district, one or two lab technicians collected the water samples, transported the samples to the laboratory in their town, conducted the tests, and recorded the results in a logbook. The lab technicians then entered these data into a digital spreadsheet or document file and forwarded it to the regional center via email.

Type C institutions, with their many testing locations and few sample collector/testers, struggled to consolidate data from the multiple testing sites. Each testing location under the Ugandan national supplier, for example, had slightly different reporting formats, which delayed collation and submission. Institutions without computers at their testing locations relied on vehicles to physically transport data from the testing location to the central level, which resulted in reporting delays for two MfSW program participants. 

#### 3.1.4. Type D (“Independent Teams”): Multiple Testing Locations, Many Collectors and Testers

Type D institutions have multiple testing locations with many individuals at each site who collect and test water samples and periodically collate the data from all locations. This typology was the least common and included one DHO in both Kenya and Uganda and the national surveillance agency in Senegal. All of these institutions tested samples using portable kits set up in offices. For example, in the Kenyan DHO, Community Health Workers (CHWs) from four sub-areas collected samples, transported them to the testing location at a local health center, and tested them while supervised by Community Health Extension Workers (CHEWs). The CHEWs recorded the results on paper log sheets and transported these sheets to the district hospital via motorbike for compilation and digital entry (Figure 4b).

Since Type D institutions have numerous collectors, testers, and testing locations, this typology presents the most challenging data management scenario. As with Type C institutions, one of the main challenges for Type D institutions was ensuring data consistency across the multiple testing locations as well as within testing locations. To address this challenge, the Kenyan DHO required all CHWs within a testing location to process samples together, which simplified both quality control and data collection. In addition, it was difficult for these institutions to collate results from the various testing locations, which frequently occurred via the transportation of paper copies by vehicle.

### 3.2. ICT applications in Water Quality Monitoring

**Table 3:** Information and communication technology (ICT) applications in water quality monitoring programs.

	Mobile Phones	Computer	Internet	GIS
Field collection	calls (coordinate sampling)	print-outs of sampling forms	NR	GPS units
Lab testing	calls and SMS messages (to consumers/operators)	NR	NR	NR
Internal reporting/conducting testing	calls and SMS (check results, clarify issues, reminders, inventory)	text editor (raw data, reports); spreadsheet software and databases (raw data); flash drives (data)	email (raw data, reports)	NR
External reporting	calls (report results)	text editor, presentation software (summaries and reports)	website (public data); email (raw data, reports)	NR
Managing sampling points	NR	Spreadsheet or text editor (customer database)	NR	GIS (maps, customer database)

NR: Use not reported among study institutions.

We documented a range of ICTs that were used by MfSW program participants in their water quality monitoring programs (Table 3): mobile phones (calls or SMS messages to coordinate programs and ad-hoc transfer of results), computers (data entry, used by two-thirds of MfSW applicants), internet (data transfer), flash drives (data transfer), and GPS (recording sampling locations and mapping water points). Across all typologies, we observed common challenges with electronic data management: computers were often unavailable or shared between multiple departments (particularly among surveillance agencies) and both urban and rural institutions often struggled with intermittent internet, loss of flash drives, frequent power outages, power surges that destroyed equipment, and viruses that corrupted files. 

Our analysis of testing program typologies in sub-Saharan Africa shows that regulated microbial water quality testing is almost always performed in dedicated spaces rather than at the water source. While in general this practice promotes centralized data entry on computers and transmission via the internet, mobile phone-based data management applications are receiving increasing attention as tools for improving information flows. To evaluate the potential for mobile phone tools to strengthen regulated water quality monitoring in Africa, we examined data flow challenges and ICT opportunities in the four institutional typologies. 

Among Type A and Type B institutions, water quality information is collected at a central location, which reduces the requirements for constant data transfers. These institutions are most likely to benefit from ICT tools that include GIS information for sample collection, electronic water quality databases, and reliable internet services for transmitting information to external agencies (e.g., Ministry of Health, regulators). To reduce sample-handling errors, Type B institutions may also benefit from bar code labeling of water samples. Type C and Type D institutions must collate data from multiple testing locations; mobile phone data applications could improve their data flows by standardizing and automating this process. In particular, the numerous sample collectors, testers, and locations in Type D institutions make it difficult to standardize and manage data collection within and between testing locations. Additionally, mobile phone data applications may be particularly useful for surveillance agencies: surveillance staff visit different water sources each time they collect water samples, often collecting significant source-related information during sampling (e.g., source type, sanitary status). In contrast, water suppliers generally collect samples from constant points within their distribution systems.

In summary, our research suggests that mobile phone-based water quality data management applications will provide minimal benefits for Type A or B institutions, but prove worthwhile for Type C institutions in some contexts. They are most likely to benefit Type D surveillance agencies. While program structure is only one component of context (for example, network connectivity and user literacy are also important), we posit that program structure is an attribute that is relatively straightforward to assess and provides a starting point for identifying when and where mobile phone applications might improve data collection and management.

### 3.3. Identifying a Context for Mobile Phone Data Collection: SNH in Senegal

**Figure 5:**
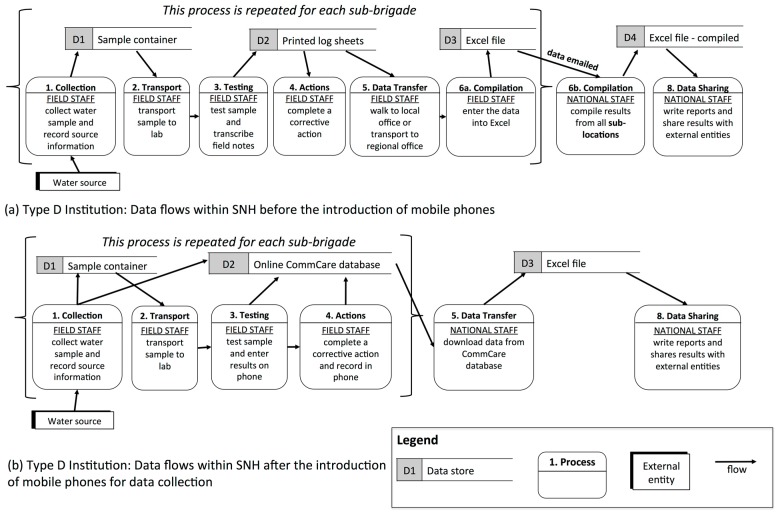
Data flow diagram for SNH. Simplified data flow diagram for Type D institution, SNH, before (**a**) and after (**b**) mobile phone intervention.

To evaluate mobile phone applications for water quality data management in a Type D context, we developed a mobile phone-based application for SNH in Senegal, which faced the following data related challenges among their 13 testing locations (‘sub-brigades’) in their water monitoring program (Figure 5):

- Health agents from different sub-brigades recorded different types of data about water sources, making it difficult to compare information across sub-brigades.
- Among sub-brigades with access to computers, data were often transcribed from paper by administrative staff and sent electronically to regional or national managers. In areas without computers or internet, agents sent data logbooks in a vehicle to regional or national offices for entry.
- Sub-brigade managers were often relocated yearly and rarely reviewed historical data, which was usually stored in paper formats.

Between August–November 2014, we trained 81 health agents from 13 sub-brigades in three regions of Senegal (Kaolack, Diourbel, and Dakar) to use the application. Through this training process and our analysis of their application use, we identified the following lessons:

- Benefits were limited by infrequent water testing. Phone usage was restricted to managing water quality data to reduce misuse; however, since rural sub-brigades only tested a few days per month, the phones were often not in use. Among health agents for whom water quality is only one of many responsibilities, the cost-effectiveness of mobile phone data collection may improve if the applications are used for programs beyond water quality.
- Efficient workflows should be developed first. While the first two data collection forms of the application specified information that the health agents already recorded (e.g., water source types, water testing results), introducing queries for new types of information, such as recording actions taken in response to poor water quality, required in-depth discussions to standardize a consistent workflow across the sub-brigades.

- Supportive institutional structures are important for ensuring sustainability. Despite frequent staff transfers, the strong social infrastructure within SNH facilitated efficient engagement of new health agents with the mobile application.
- Mobile phone literacy is high. Few of the health agents were experienced with computers, but all were adept at manipulating mobile phones.
- Even within the same institution, support requirements and available resources vary. For example, in urban Dakar, sub-brigades included administrative and IT support staff that were not available to rural sub-brigades.

## 4. Conclusions

Through a study of water quality monitoring programs among 26 water suppliers and public health agencies across six African countries, we identified four main program structures or typologies, which we have termed A through D. Though most of the institutions struggled to access data for both immediate action and long-term decisions, our analysis of data flows within each of the four typologies suggests that mobile phone-based water quality data management applications will provide minimal benefits for Type A or B institutions, may prove worthwhile for Type C institutions in some contexts, and are most likely to benefit Type D surveillance agencies, which rely on numerous sample collectors and testers to monitor a variety of sources and locations in multiple remote locations.

The popularity of mobile phone data management applications has fostered the perception that all field-based data collection activities will benefit from their use. In the case of regulated water quality monitoring in Africa, however, our results show that water quality testing is almost always conducted in laboratory or office facilities—not at the water source. This practice promotes data entry on computers and transmission via the internet, which is often accessed through a broadband connection. After analyzing and classifying the common water testing structures found among African water suppliers and surveillance agencies, we propose that mobile phone applications will provide the greatest efficiency gains in water quality data management among institutions that collect water quality data from many remote laboratories that are not equipped with computers, *i.e.* where smart phones are cost-effective alternatives to computers.

Our implementation of a mobile phone application in one such setting highlights another feature of water quality monitoring that presents a challenge for phone-based data management in remote settings: water sample collection and testing can be relatively infrequent and may only be conducted by a few individuals at a health center or water treatment facility. As a result, staff may forget how to use dedicated phone applications for data entry and submission. Furthermore, the cost-effectiveness of the training and phone supply programs is low with respect to actual data transmission. In the case of surveillance agencies with multiple public health responsibilities, both of these issues might be addressed by utilizing mobile phone applications (possibly the same one) for multiple data collection activities. Limited testing can also make it difficult for agents to maintain their proficiencies in water-testing procedures. Developing mobile phone applications for re-training agents could help maintain their water quality testing performance. Additionally, managing inventory, including equipment or consumable goods, was another challenge identified during our implementation that could be addressed by future iterations of the application.

The four water quality testing typologies that we describe in this study are based on an analysis of 26 African institutions that applied for participation in MfSW and may not be representative of all water quality information flows. However, the 26 MfSW participants include water suppliers and surveillance agencies from six African countries, which suggest that we have described the most common typologies, which provide reference points for additional classification. Future studies could test the hypothesis that mobile phones will provide little benefit for Type A and B institutions, but may provide some benefits for Type C institutions.

Finally, this paper addresses information flows from the water source to a central, collated format within an organization. It is important to note that these are the first steps in data management systems for water quality monitoring programs. Further research is needed to determine which ICT-based interventions will prove most efficient for additional reporting, including the transmission of water quality data to both regulatory authorities and the public. 
